# Amino Acid-Based Metabolic Panel Provides Robust Prognostic Value Additive to B-Natriuretic Peptide and Traditional Risk Factors in Heart Failure

**DOI:** 10.1155/2018/3784589

**Published:** 2018-10-10

**Authors:** Chao-Hung Wang, Mei-Ling Cheng, Min-Hui Liu

**Affiliations:** ^1^Heart Failure Research Center, Division of Cardiology, Department of Internal Medicine, Chang Gung Memorial Hospital, Keelung, Chang Gung University College of Medicine, Taoyuan, Taiwan; ^2^Metabolomics Core Laboratory, Healthy Aging Research Center, Chang Gung University, Taoyuan, Taiwan; ^3^Department and Graduate Institute of Biomedical Sciences, College of Medicine, Chang Gung University, Taoyuan, Taiwan; ^4^Clinical Metabolomics Core Laboratory, Linkou Chang Gung Memorial Hospital, Taoyuan, Taiwan; ^5^Department of Nursing, National Yang-Ming University, Taipei, Taiwan

## Abstract

Metabolic disturbances represent functional perturbation in peripheral tissues and predict outcomes in patients with heart failure (HF). This study developed an amino acid-based metabolic panel and sought to see whether this panel could add diagnostic and prognostic value to currently used B-type natriuretic peptide (BNP) measurements. Mass spectrometry and ultra-performance liquid chromatography were performed on 1288 participants, including 129 normal controls and 712 patients at HF stages A to D in the initial cohort and 447 stage C patients in the validation cohort. Patients were followed up for composite events (death/HF-related rehospitalization). Histidine, ornithine, and phenylalanine were 3 metabolites found strongly significant to identify patients at stage C and were adopted to develop the HOP panel. Compared to BNP, HOP had better value in discriminating the patients at different stages, especially in elderly patients and those with atrial fibrillation, high body mass index, or kidney dysfunction. HOP was correlated with the distance of 6 min walking distance better than BNP. For prognosis, HOP predicted composite events in patients at stages C and D, independent of log (BNP), age, sex, left ventricular ejection fraction, New York Heart Association functional class, HF stage, diabetes mellitus, chronic kidney disease, hypertension, hemoglobin, and albumin. Higher BNP (≥750 pg/mL) along with higher HOP (≥14) robustly predicted lower event-free survival compared to all others [hazard ratio = 3.15 (2.23–4.46), *p* < 0.001]. The prognostic value of HOP was confirmed in the validation cohort. In conclusion, aiming for clinical applications, this study proved that the HOP panel provides diagnostic and prognostic value additive to BNP and traditional risk factors.

## 1. Introduction

Metabolomics is a rapidly growing field with the potential to provide a comprehensive molecular landscape for describing metabolic health, tracking response to treatment, and monitoring disease recurrence [1]. A number of metabolomics studies of patients with HF have provided novel metabolic panels for diagnosis and prognosis [2–5]. Metabolic disturbances identified by metabolomics in plasma represent functional perturbation in peripheral tissues and potentially compensate for the value of B-type natriuretic peptide (BNP), which majorly indicates cardiac wall stress [6, 7].

Metabolites applied for clinical diagnosis and prognosis included amino acids and a variety of lipids [1–5]. Although metabolomics provides complimentary information on metabolism, it is not applicable for clinical use at current status. Considerable practical issues include the availability of mass spectrometry, stability of measuring low concentration metabolites, and interpretation of sophisticated metabolite profiles. Amino acids are metabolites that are feasible to quantify by ultra-performance liquid chromatography (UPLC) available worldwide. Targeting amino acids with relatively high plasma concentrations and measurable by UPLC offers a promising solution. To establish a clinically applicable metabolite panel, the aims of this study included the following: (1) based on the dataset of normal controls and patients at stage C HF (according to the American College of Cardiology and the American Heart Association HF classification system) [8], to identify the potential diagnostic metabolites from high concentration amino acids and develop a UPLC-based amino acid panel; (2) to test the ability of this panel in discriminating different stages of HF; and (3) based on the dataset from HF patients with potential of events (at HF stages C and D), to see whether this panel could improve prognostic value. The prognostic value was further tested in an independent validation cohort.

## 2. Methods

### 2.1. Patients and Study Design

For the initial study, we enrolled patients at HF stages A to D and normal controls from January 2011 to May 2014. HF stages A to D were classified according to the American College of Cardiology and the American Heart Association HF classification system [8]. Patients at stage C were those hospitalized due to acute or decompensated chronic HF and aged 20–85 years, with a left ventricular ejection fraction (LVEF) < 50%. Patients at stage D were those with marked symptoms after receiving optimal medical therapy. Patients at stage B were asymptomatic and included postacute myocardial infarction patients regardless of their LVEF, those with any severe structural abnormalities, and those with an LVEF of <50%. Patients at stage A included (i) asymptomatic patients with risk factors, with or without angiogram-documented coronary disease, and with an LVEF of >50% and (ii) those without structural heart disease. Meanwhile, normal controls were aged 20–85 years and had no significant systemic disease, such as hypertension, diabetes mellitus, or coronary artery disease. They were not on any medications and had an LVEF of >60%.

Exclusion criteria included (i) the presence of systemic diseases such as hypothyroidism, decompensated liver cirrhosis, and systemic lupus erythematosus; (ii) the presence of disorders other than HF that might compromise survival within 6 months; (iii) patients being bedridden for >3 months and/or unable to stand alone; (iv) patients with a serum creatinine of >3 mg/dL; and (v) patients with severe coronary artery disease without complete revascularization therapy. Informed consent was obtained from all patients. The study was designed and carried out in accordance with the principles of the *Declaration of Helsinki* and with approval from the Ethics Review Board of Chang Gung Memorial Hospital.

To validate the prognostic value of the metabolic panel, a second independent cohort, including 447 patients at stage C as defined above, was recruited from July 2013 to January 2016. (The study flow diagram is provided in Supplementary Figure I.)

### 2.2. Blood Sampling and Examination

For patients at stages C and D, blood samples for metabolomics were collected in the early morning after fasting for 8 hours before discharge from the hospital in EDTA-containing tubes. Plasma was analyzed by metabolomics workflow. BNP was measured with the Triage BNP Test (Biosite, San Diego, CA), which was a fluorescence immunoassay for quantitative determination of plasma BNP. Precision, analytical sensitivity, and stability characteristics of the assay were previously described [6]. For normal controls and patients at stages A and B, blood samples for metabolomics analysis and BNP were collected at enrollment. Measurement of other parameters, including kidney function, hemoglobin, *γ*-glutamyltransferase (*γ*GT), aspartate aminotransferase (AST), alanine aminotransferase (ALT), estimated glomerular filtration rate (eGFR), and albumin, was conducted in the central core laboratory.

### 2.3. Follow-Up Program

For patients at stages C and D, follow-up data were prospectively obtained every month from hospital records, personal communication with the patients' physicians, telephone interviews, and patients' regular visits to staff physician outpatient clinics for 3 years. “Rehospitalization” was defined as HF-related rehospitalizations. A committee of 3 cardiologists adjudicated all hospitalizations without knowledge of patients' clinical variables to determine whether the events are related to worsening HF. “All-cause death” was chosen as an endpoint because of the interrelationship of HF with other comorbidities in the patient cohort. Only was the composite event of HF-related rehospitalization and all-cause death analyzed for the prognostic purpose. In the second independent population for validation, patients were followed up for one year.

### 2.4. Stable Isotope Dilution-Multiple Reaction Monitoring Mass Spectrometry

The metabolomic analyses were carried out with the AbsoluteIDQ® p180 Kit (Biocrates Life Sciences AG, Innsbruck, Austria). The kit enables us to identify and quantify 21 amino acids. All reagents used in this analysis were of LC-MS grade. Ten *μ*L aliquot of each plasma sample was mixed with isotopically labeled internal standards in a multititer plate and dried under nitrogen. Amino acids were derivatized with 5% phenyl isothiocyanate for 20 min and subsequently dried under nitrogen. Three hundred *μ*L of extraction solvent (5 mM ammonium acetate in methanol) was added, and after 30 min incubation, it was centrifuged for 2 min at 100 × g. Subsequently, 150 *μ*L of filtrate was transferred to a microtiter plate and diluted with 150 *μ*L of water for analysis of amino acids by LC-MS/MS. The remaining filtrate was mixed with 400 *μ*L of running solvent for flow injection analysis coupled with tandem mass spectrometric analysis (FIA-MS/MS). The analysis was performed in positive and negative electrospray ionization mode. Identification and quantification were achieved by multiple reaction monitoring. It was standardized by spiking in of isotopically labeled standards. LC-MS analysis was performed with Waters Xevo TQ coupled to a UPLC (Waters Corp., Milford, USA). Metabolites were separated on a reversed-phase column (2.1 mm × 50 mm, BEH C18, Waters Corp., Milford, USA) using a mobile phase, which was composed of a gradient mixture of solvent A (formic acid 0.2% in water) and solvent B (formic acid 0.2% in acetonitrile) (0 min 0% B, 3.5 min 60% B, 3.8 min 0% B, and 3.9 min 0% B). Elution was performed at a flow rate of 900 *μ*L/min. The column temperature was maintained at 50°C. For FIA, an isocratic method was used (100% organic running solvent) with varying flow conditions (0 min, 30 *μ*L/min; 1.6 min, 30 *μ*L/min; 2.4 min, 200 *μ*L/min; 2.8 min, 200 *μ*L/min; and 3 min 30 *μ*L/min). The corresponding MS settings were as follows: dwell time of 0.019–0.025 sec, capillary voltage at 3.92 KV for positive mode, capillary voltage at 1.5 kV for negative mode, nitrogen as collision gas medium, and source temperature at 150°C.

### 2.5. Ultra-Performance Liquid Chromatography- (UPLC-) Based Measurement

The plasma samples (100 *μ*L) were precipitated by 10% sulfosalicylic acid. After protein precipitation and centrifugation, derivatization was initiated by AQC in acetonitrile. Then, amino acids were analyzed using the ACQUITY UPLC System consisted of a Binary Solvent Manager, a Sample Manager, and a Tunable UV detector. The system was controlled and data collected using Empower™ 2 Software. Separations were performed on a 2.1 × 100 mm ACQUITY BEH C18 column at a flow rate of 0.70 mL/min. The average intra-assay coefficient of variation was 4.3% for histidine, 4.6% for ornithine, and 4.6% for phenylalanine. A total coefficient of variation was 3.1% for histidine, 3.6% for ornithine, and 3.7% for phenylalanine. The detection limit was 0.5 *μ*M for histidine, 2.0 *μ*M for ornithine, and 3.3 *μ*M for phenylalanine. The linear range was 25–500 *μ*M for these four amino acids. Spermidine level was low with high coefficient of variation. Lipid metabolites were not measurable by UPLC.

### 2.6. Measuring Skeletal Muscle Mass

Segmental multifrequency bioelectrical impedance analysis (SMBIA) was used to measure skeletal muscle mass. Eight-polar bioimpedance analyses of each participant were obtained using an InBody 720 multifrequency analyzer (at 1, 5, 50, 250, 500, and 1000 kHz) (Model Biospace InBody 720, Seoul, Korea). The validity of SMBIA has been documented in previous studies [9–11], showing no statistical difference between magnetic resonance imaging-measured and SMBIA-derived values for skeletal muscle mass. Skeletal muscle mass was measured for patients at stage C on the day we collected blood samples and 2 weeks later. The difference between the two measurements was correlated to the metabolic data. Skeletal muscle growth and loss were defined as skeletal muscle mass at two weeks minus skeletal muscle mass at baseline, with >0 kg signaling growth and <0 kg signaling loss.

### 2.7. Six-Minute Walking Distance

To estimate the patients' functional condition, we measured six-minute walking distance (6MWD) for patients at stages ranging from B to D on the day metabolites were analyzed. Exclusion criteria were (1) extremity problems (polio, arthralgia, and amputation, *n* = 43), (2) deafness (*n* = 4), (3) uncontrolled chronic pulmonary diseases (*n* = 14), (4) age > 85 years old (*n* = 5), (5) severe obesity (*n* = 31), (6) post-surgery with wound pain (*n* = 8), (7) uncontrolled psychiatric problems (*n* = 3), (8) uncorrected congenital heart disease (*n* = 5), (9) not able to walk (*n* = 12), and (10) stroke with hemiparesis (*n* = 41). Finally, 6MWD was performed in 453 patients.

### 2.8. Statistical Analysis

Results are expressed as mean ± SD for continuous variables and as the number (percentage) for categorical variables. Data were compared by two-sample *t*-tests, ANOVA (subgroup analysis was conducted by Bonferroni), and chi-square (multiple comparison with Bonferroni adjusted *p* value), when appropriate. The linear trend of the distribution of demographic and laboratory data across study groups was tested using Cochran-Armitage chi-square analysis for categorical variables and linear contrast in the general linear model for continuous variables. For accuracy measurement, a multinomial logistic regression model was used. The classification accuracy was calculated by comparing the predicted probability of HOP score and log (BNP) based on the logistic model to the dependent variable defined by different HF stages. For subgroup analysis, the cutoffs of BMI and eGFR were set at 24 kg/m^2^ (mild obesity) and 60 mL/min/1.73 m^2^ (≥stage 3 chronic kidney disease), respectively. Spearman's correlation analysis was used to assess the correlation between variables. The integrated MetIDQ software (Biocrates, Innsbruck, Austria) was applied to streamline data analysis by automated calculation of metabolite concentrations. To maximize identification of differences in metabolic profiles between groups, the OPLS-DA model was applied and performed using the SIMCA-P software (version 13.0, Umetrics AB, Umea, Sweden). The variable importance in the projection (VIP) value of each variable in the model was calculated to indicate its contribution to the classification. A higher VIP value represents a stronger contribution to discrimination between groups. The VIP values of those variables greater than 1.0 are considered significantly different. For diagnosis, stepwise logistic regression analysis was used. Variables with VIP > 1 and *p* value of <0.05 in the univariate analysis were selected for the multivariable analysis. Odds ratios (ORs) and 95% confidence intervals (CIs) were calculated. The HOP score was calculated by a combination of histidine, ornithine, and phenylalanine according to the formula *β*_1_ *X*_1_ + *β*_2_ *X*_2_ + *β*_3_ *X*_3_, with *Xj* denoting the standardized value for the *j*th metabolites and *βj* denoting the regression coefficient from the regression model containing the indicated metabolites. Receiver operating characteristic (ROC) curves and area under curves (AUCs) were also estimated, and the cutoff value of variables was identified based on Youden's index. Follow-up data were collected as scheduled or at the last available visit. Cox proportional hazards models were used to determine independent predictors of the first defined events (death, or HF-related rehospitalization). Variables with *p* value <0.05 in the univariate analysis were selected for the multivariable analysis. Hazard ratios (HRs) and 95% CIs were also calculated. All statistical analyses were 2-sided and performed using SPSS software (version 22.0, SPSS, Chicago, IL, USA). A *p* value of <0.05 was considered significant.

## 3. Results

### 3.1. Baseline Characteristics

A total of 841 participants, including 129 normal controls and 712 patients at HF stages A (*n* = 93), B (*n* = 120), C (*n* = 398), and D (*n* = 101), were enrolled in the initiation cohort. Baseline characteristics and laboratory data are shown in Table 1. From stage A to stage D, patients had a trend to be older and have lower LVEF, blood pressure, cholesterol, triglyceride, hemoglobin, albumin, and eGFR, but have higher heart rate, BNP levels, and incidence of diabetes mellitus, chronic kidney disease, and atrial fibrillation. The main etiology of HF in the study cohort was coronary artery disease.

### 3.2. Development of Amino Acid-Based Metabolic Panel

**Figure 1:**
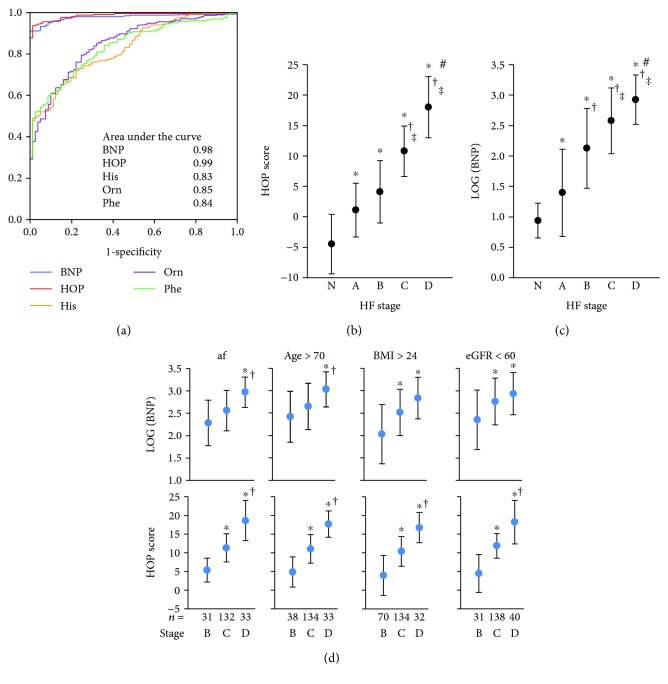
Diagnostic value of amino acid-based metabolic profile and B-natriuretic peptide (BNP). (a) The receiver operating characteristic curves and area under the curves. (b and c) The HOP and log (BNP) at different heart failure (HF) stages. ^∗^ *p* < 0.05, compared to normal controls (N); ^†^ *p* < 0.05, compared to stage A; ‡*p* < 0.05, compared to stage B; ^#^ *p* < 0.05, compared to stage C. (d) Log (BNP) and HOP in patients with atrial fibrillation (af), age > 70 years, body mass index (BMI) > 24 kg/m^2^, and estimated glomerular filtration rate (eGFR) < 60 mL/min/1.73 m^2^. ^∗^ *p* < 0.05, compared to stage B; ^†^ *p* < 0.05, compared to stage C. HOP: a metabolic panel composed of histidine, ornithine, and phenylalanine.

Comparisons between normal controls and stage C patients in all essential and nonessential amino acids measure by mass spectrometry are shown in Table 2. To identify a potential amino acid panel for diagnosing HF, we performed multivariable analyses only for the amino acids with a VIP score > 1. Histidine, ornithine, and phenylalanine were three strongly and independently significant amino acids for the integrated metabolic panel and were finally adopted to develop the HOP panel based on the logistic regression model. For unified quantification, all HOP measurements in subsequent studies were performed by UPLC. Histidine, ornithine, and phenylalanine changed in a significant trend from normal patients to stages A through D (Table 1). The diagnostic value of the HOP and log (BNP) for discriminating stage C patients from normal controls are shown by the ROC curves and AUCs (Figure 1(a)) and by univariate analysis in Table 3. The HOP gave rise to an AUC of 0.99, similar to BNP (AUC = 0.98). In multivariable analysis, the diagnostic value of the HOP and BNP was independent of age, sex, left ventricular ejection fraction, diabetes mellitus, kidney disease, and hypertension (Table 3). In stage C patients, HOP scores were not significantly different when comparing those taking angiotensin-converting enzyme inhibitors/angiotensin receptor blockers and those who did not (10.7 ± 4.13 vs. 11.3 ± 4.14, respectively, *p* = 0.42). The difference between patients taking *β*-blockers and those who did not also did not reach the level of significance (10.6 ± 4.01 vs. 11.3 ± 4.46, respectively, *p* = 0.15).

### 3.3. HOP and BNP in Discriminating HF Stages

HOP and log (BNP) significantly separated patients at different HF stages (Figures 1(b) and 1(c)). The accuracy of HOP in discrimination was better than that of log (BNP) (66.2% and 59.4%, respectively). In patients at stages B, C, and D, the HOP discriminated stages B and C better than log (BNP) in elderly patients aged > 70 years (79.9% and 66.8% accuracy, respectively) and those with atrial fibrillation (78.2% and 69.8% accuracy, respectively) (Figure 1(d)). The HOP also demonstrated better discrimination between stages C and D than log (BNP) in patients with a body mass index > 24 kg/m^2^ (73.7% and 62.3% accuracy, respectively) and eGFR <60 mL/min/1.73 m^2^ (79.9% and 65.0% accuracy, respectively).

### 3.4. Correlating the HOP Panel to Clinical Variables

HOP showed modest correlation with BNP levels (Supplementary Figure IIA). Ornithine levels were weakly associated with the levels of *r*GT (*r* = 0.33, *p* < 0.001), but not with ALT or AST. Although both *r*GT and ornithine levels changed in a significant trend from normal patients to stages A through D, the areas under ROC curves in discriminating stage C patients from normal controls were 0.71 and 0.85, respectively. Skeletal muscle growth and loss were significantly related to baseline phenylalanine levels (Supplementary Figure IIB) and weakly related to BNP (*r* = −0.39, *p* < 0.001). In patients who lost skeletal muscle, skeletal muscle mass changed from 29.9 ± 6.11 kg at baseline to 28.6 ± 5.69 kg (*p* < 0.001) two weeks later. Patients who experienced skeletal muscle growth progressed from 28.1 ± 4.17 kg to 28.9 ± 4.13 kg two weeks later (*p* < 0.001). Baseline phenylalanine levels were significantly higher in patients who had skeletal muscle loss compared to those who had skeletal muscle growth (86.5 ± 22.8 *μ*M vs. 68.6 ± 12.5 *μ*M, respectively, *p* < 0.001). To estimate the correlation between changes in phenylalanine and changes in skeletal muscle mass, we had 101 patients with blood samples also collected two weeks after baseline. To discriminate patients who experienced muscle growth from those who experienced muscle loss, we used ROC curves to determine the optimal baseline phenylalanine cutoff value (69.5 *μ*M), with high phenylalanine levels defined as ≥69.5 *μ*M and low phenylalanine levels as <69.5 *μ*M. Using baseline phenylalanine and phenylalanine measurements taken two weeks later, we classified patients according to the following patterns: “high-to-high,” “high-to-low,” “low-to-low,” and “low-to-high.” The skeletal muscle loss was significantly greater in high-to-high patients, compared to the others (Supplementary Table I). For functional assessment, 6MWD demonstrated a significant correlation with HOP that was stronger than with BNP (Supplementary Figures IIC and IID), but not with albumin. Higher HOP was associated with shorter 6MWD.

### 3.5. Prognostic Value of HOP

The prognostic value of HOP was further tested in patients with potential risk of events. During a follow-up period of three years, we observed 172 (34.5%) composite events (death/HF-related rehospitalization) in patients at stages C and D (*n* = 499). In univariate analysis, associates of composite events included HOP, log (BNP), age, sex, left ventricular ejection fraction, functional class, HF stage, diabetes mellitus, chronic kidney disease, hypertension, hemoglobin, and albumin (Table 4). In multivariable analysis, HOP remained significant after adjusting for log (BNP), age, sex, left ventricular ejection fraction, functional class, HF stage, diabetes mellitus, chronic kidney disease, hypertension, hemoglobin, and albumin. However, log (BNP) became insignificant (Table 4).

**Figure 2:**
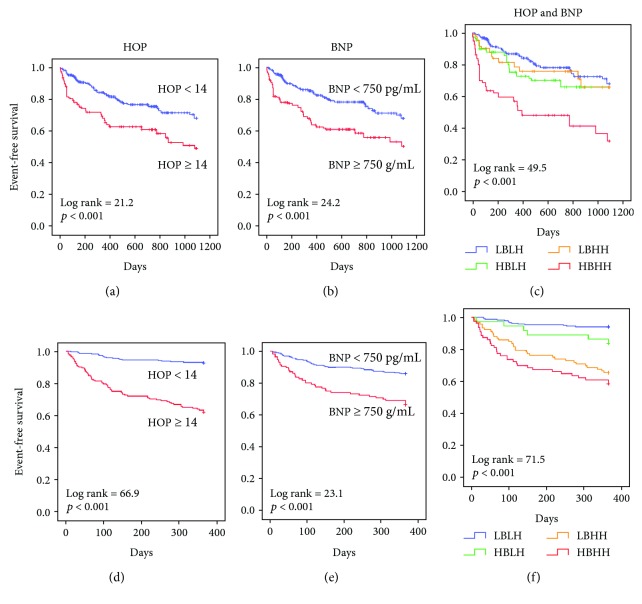
Prognostic value by combining B-natriuretic peptide (BNP) and HOP. Kaplan-Meier curves of HOP and BNP in the initiation (a and b) and the validation cohorts (d and e); the Kaplan-Meier curves of 4 subgroups in the initiation (c) and the validation cohorts (f), including high BNP and HOP (HBHH), high BNP and low HOP (HBLH), low BNP and high HOP (LBHH), and low BNP and low HOP (LBLH). High and low BNP are defined by BNP ≥ 750 and <750 pg/mL, respectively. High and low HOP are defined by HOP ≥ 14 and <14, respectively. HOP: a metabolic panel composed of histidine, ornithine, and phenylalanine.

The cutoff values of BNP and HOP were set at 750 pg/mL and 14 based on ROC curves. Kaplan-Meier curves show that patients with HOP ≥ 14 had lower 3-year event-free survival compared to those with HOP < 14 (Figure 2(a)). Patients with BNP ≥ 750 pg/mL had lower event-free survival compared to those with BNP < 750 pg/mL (Figure 2(b)). Based on the cutoffs, patients were separated into 4 subgroups, including high BNP and HOP (HBHH), high BNP and low HOP (HBLH), low BNP and high HOP (LBHH), and low BNP and low HOP (LBLH). Kaplan-Meier survival curves were further analyzed according to the 4 subgroups (Figure 2(c)). Patients with HBHH had significantly lower 3-year event-free survival than all others (HR = 3.15, 95% CI = 2.23–4.46, *p* < 0.001). The prognostic value of the HOP panel was further validated in 447 stage C patients. During the one-year follow-up period, 85 (19%) composite events were noted. Patients with HOP ≥ 14 (Figure 2(d)) and those with BNP ≥ 750 pg/mL (Figure 2(e)) were associated with a higher event rate over one year from enrollment. Those with HBHH also had the lowest one-year event-free survival rate than all others (Figure 2(f)). The demographic data for the validation cohort are shown in Supplementary Table II.

### 3.6. Differences between Subgroups Defined by BNP and HOP

Table 5 shows the differences in demography and laboratory data between four subgroups. Compared to patients with LBLH, those with HBHH had higher incidence of ischemic etiology, and higher levels of total bilirubin, but lower left ventricular ejection fraction, lower high-density lipoprotein cholesterol levels, serum sodium, hemoglobin, albumin, and eGFR. Patients with LBHH, compared to those with LBLH, had higher levels of total bilirubin, but lower levels of total cholesterol and high and low density of lipoprotein-cholesterol and albumin. Patients with HBHH, compared to those with HBLH, had lower levels of sodium and albumin. Compared to patients with LBHH, patients with HBHH had higher incidence of ischemic etiology and higher levels of low-density lipoprotein cholesterol, but they also had lower incidence of atrial fibrillation as well as lower left ventricular ejection fraction, body mass index, hemoglobin, albumin, and eGFR.

## 4. Discussion

The UPLC-based amino acid panel provided a metabolism spectrum for patients with HF. In identifying stage C patients, HOP was not inferior to BNP. However, HOP discriminates HF stages better than BNP especially in the elderly and those with atrial fibrillation, high body mass index, or kidney dysfunction. HOP was significantly associated with losses of both skeletal muscle mass and functional capacity. HOP added diagnostic and prognostic value to currently used BNP and a variety of traditional risk factors. Combining HOP and BNP synergistically and robustly identified patients at high risk of events.

### 4.1. Issues Relating to Current Biomarkers

BNP is a powerful diagnostic biomarker for HF, with ST2 and galectin 3 providing additional prognostic information [6, 12, 13]. However, all these biomarkers offer limited clues for aspects other than wall stress and activation of global fibrosis. The clinical benefit of natriuretic peptide-guided therapy has not been uniform across the trials, with some populations not shown benefit [7]. Although to define more precise and clinically attainable natriuretic peptide targets is suggested, this is complicated in the presence of the common confounders such as atrial fibrillation, ischemia, aging, and renal dysfunction [7]. Our study showed that HOP added diagnostic value to BNP. HOP offers a quantifiable readout of the biochemical state of metabolism in HF-related end organs that is often not obvious from BNP analysis. It seems that BNP represents the wall stress of the heart, which is central, while HOP represents the global metabolism status of peripheral tissues.

The relationship between BNP and HOP is only modest. Notably, the correlation between HOP score and BNP in the zone of BNP < 500 pg/mL or > 5000 pg/mL was better than that in the zone of BNP from 500 to 5000 pg/mL (Supplementary Figure IIA). Level of stress on cardiac chambers is apparently different from the peripheral metabolism status associated with the imbalance between demand and supply. Patients with high BNP were not always at high risk of events. High BNP with low HOP represented increased ventricular wall stress, but without significant impact of perturbed HF-related hemodynamics on peripheral tissues. Patients with both high BNP and high HOP were at very high risk of both central and peripheral disturbances. Compared to other subgroups, these patients tended to have lower ejection fractions, as well as lower levels of albumin, hemoglobin, sodium, high-density lipoprotein cholesterol, and eGFR. They also had higher incidence of ischemic etiology and higher total bilirubin levels. This subgroup had an event-free survival rate of only 51% one year after discharge. On the other hand, although patients with high HOP but low BNP had event risk similar to those with low BNP and low HOP, this subgroup represented a population with metabolism dysfunction and malnutrition, indicated by lower lipid profile and albumin levels, and demanded interventions designed specifically.

### 4.2. HOP Provides Insight into Metabolism

HOP is developed from statistical integration of molecular-level metabolites with no prior comprehension of the exact relevance of each component. Nitric oxide synthesis dysregulation and tetrahydrobiopterin depletion were probably related to impaired conversion of phenylalanine to tyrosine and the accumulation of phenylalanine [14, 15]. On the other hand, the increased phenylalanine levels may also be associated with increased muscular protein breakdown [16] and impaired liver function. Although our data revealed that high phenylalanine levels at baseline or persistently high phenylalanine levels are modestly correlated with skeletal muscle mass loss, the formal evaluation of muscle protein dynamics involves sophisticated calculation based on isotope infusion designs [17, 18] and is impractical for HF patients. The kinetics of circulating phenylalanine waits for further investigation and clarification in the future.

A large amount of histidine pooled in hemoglobin and carnosine in the muscle, delaying indications of histidine deficiency [19]. Consequently, by the time lower blood histidine concentration was noted, the histidine deficiency was probably advanced. For the decreased concentration of histidine, there are a few speculative mechanisms. First, in normal physiology, the protein turnover rate is much higher than that of carbohydrates and fat [18]. However, a majority of the broken proteins are efficiently resynthesized to form new proteins. Once histidine is used to synthesize muscle, it turns into another form, namely, methylhistidine [17, 18]. Based on the high turnover rate in muscle of HF patients, a huge amount of histidine is consumed without adequate refilling of the histidine pool. Second, histidine can be converted to glutamate and enter the glutamate-ornithine-proline pathway or Krebs cycle to produce energy for cardiac tissues. Finally, glucose is able to be converted to phosphoribosyl pyrophosphate for histidine biosynthesis. Overuse of glucose to produce energy in HF impairs the production of histidine. In sum, there is a strong possibility of unbalanced histidine supply and metabolism. However, again, identifying the real mechanisms requires further investigation. At the present stage for clinical application, the changes in histidine and phenylalanine levels may serve as feedback indicators for evaluating patients' response to medications, rehabilitation, and nutritional interventions.

Some of the amino acids included in our study are not metabolized within skeletal muscle, such as ornithine and citrulline. The correlation between ornithine and traditional liver biochemistry is weak. So far, clinically available biochemical tests for liver function are neither functional nor specific. Elevated AST and ALT represent hepatocyte injury or necrosis, but are likewise insensitive for assessing HF-related liver dysfunction. *r*GT relates to liver fibrosis, but is also increased in biliary diseases and alcoholism. Ornithine is an important component of the urea cycle, which functions entirely in the liver. Ornithine level also represents the imbalance between the ornithine load derived from metabolism of amino acids and the function of urea cycle to clean ornithine. A significant portion of patients with normal *r*GT had increased ornithine levels, probably indicating acute liver congestion without established liver fibrosis. Increased *r*GT with normal ornithine may suggest a biliary or alcoholic etiology and may not necessarily be associated with HF. Although a significant trend of increase in ornithine levels from normal patients to stages A through D was noted, the relationship between ornithine and liver function indeed waits for future experiments and clinical studies. On the other hand, decreased citrulline levels have been observed in stage C patients compared to normal controls. This may be caused by impaired mitochondrial function which limits metabolism of ornithine to form citrulline in the urea cycle, or it may be caused by impaired nitric oxide synthase function [20].

### 4.3. Study Limitations

The findings in this study are limited to HF patients with LVEF < 50%. A 2-week time period to estimate the correlation between phenylalanine levels and changes in skeletal muscle mass was short. Although a longer observation time period would be more convincing, the design was substantially limited by multiple factors with potential interference, such as exercise. The effects of renin-angiotensin-aldosterone system and *β*-blockade on tissue metabolism have been investigated and remain controversial [21–23]. Although our study did not produce statistically significant findings, our results could be confounded by the presence of preexisting contraindications to using these medications. Future studies could elaborate on these findings using a randomized design or by measuring metabolites before and after receiving medication. Moreover, this study is substantially limited by the unclear relationship between amino acids and their clinical value. Majority of the findings were based on statistical observations. Future works on the mechanism exploration by cell and animal models, and clinical studies are mandatory.

## 5. Conclusion

Aiming for clinical applications, this study developed a three amino acid-based metabolic panel for assessing HF patients. The metabolic insight of HOP compensates for the value of BNP. HOP adds diagnostic and prognostic value to currently used BNP measurement and other traditional risk factors. Whether tailored multidisciplinary intervention guided by the HOP and natriuretic peptides can modify disease outcomes warrants future interventional studies.

## Figures and Tables

**Table 1:** Demographic and laboratory data in normal controls and heart failure patients at different ACC/AHA stages (Initiation cohort).

	Normal	Stage A	Stage B	Stage C	Stage D	
	*n* = 129	*n* = 93	*n* = 120	*n* = 398	*n* = 101	*p* for trend^∗^
Age (years)	53.7 ± 8.0	58.6 ± 11.4	60.9 ± 12.3	60.7 ± 14.2	61.5 ± 13.0	<0.001
Male (%)	46 (35.7)	64 (68.8)	83 (69.2)	263 (66.1)	66 (65.3)	0.465
LVEF (%)	71.1 ± 11.9	64.8 ± 15.9	46.2 ± 15.4	33.1 ± 11.3	28.7 ± 10.4	<0.001
Blood pressure (mm Hg)						
Systolic	123.2 ± 15.5	127.4 ± 17.7	124.9 ± 20.8	123.4 ± 18.5	117.2 ± 17.4	<0.001
Diastolic	74.3 ± 11.4	77.6 ± 11.0	76.1 ± 14.1	74.5 ± 12.5	73.1 ± 13.1	0.008
Heart rate, beats/min	73.1 ± 11.5	75.6 ± 13.5	74.6 ± 14.1	77.3 ± 11.8	79.4 ± 13.1	0.016
Comorbidity						
Diabetes mellitus (%)	0 (0)	30 (32.3)	45 (37.5)	156 (39.2)	46 (45.5)	0.046
Chronic kidney disease (%)	0 (0)	16 (17.2)	20 (16.7)	97 (24.4)	29 (28.7)	0.015
Hypertension (%)	0 (0)	51 (54.8)	87 (72.5)	255 (61.1)	68 (67.3)	0.287
Atrial fibrillation (%)	0 (0)	3 (3.2)	31 (25.8)	135 (33.9)	34 (33.7)	<0.001
COPD (%)	0 (0)	6 (6.5)	12 (10.0)	45 (11.3)	11 (10.9)	0.239
Ischemia (%)	0 (0)	45 (48.4)	80 (66.7)	175 (44.0)	48 (47.5)	0.059
Body mass index (kg/m^2^)	24.1 ± 3.9	25.1 ± 4.0	25.8 ± 4.4	25.2 ± 5.3	25.9 ± 7.9	0.368
Medication						
ACEI or ARB (%)	0 (0)	28 (30.1)	94 (78.3)	358 (89.9)	92 (91.1)	<0.001
*β*-Blocker (%)	0 (0)	31 (33.3)	90 (75.0)	301 (75.6)	67 (66.3)	<0.001
Diuretic (%)	0 (0)	11 (11.81)	45 (37.5)	236 (59.3)	71 (70.3)	<0.001
Laboratory data						
BNP (log)	0.94 ± 0.29	1.39 ± 0.72	2.13 ± 0.65	2.58 ± 0.54	2.92 ± 0.41	<0.001
Cholesterol (mg/dL)	211.1 ± 34.8	192.7 ± 53.2	189.4 ± 53.0	177.2 ± 45.8	157.3 ± 36.0	<0.001
Triglyceride (mg/dL)	102.2 ± 58.2	153.7 ± 102.4	142.1 ± 96.8	126.8 ± 80.2	114.5 ± 93.1	0.001
Serum sodium (mEq/L)	140.2 ± 1.1	138.6 ± 4.2	139.2 ± 2.4	139.1 ± 3.3	138.3 ± 3.3	0.468
Hemoglobin (g/dL)	13.87 ± 1.40	13.98 ± 1.68	13.55 ± 1.97	13.35 ± 2.12	12.95 ± 2.06	<0.001
Albumin (g/dL)	4.29 ± 0.72	4.05 ± 0.59	3.80 ± 0.49	3.61 ± 0.47	3.32 ± 0.51	<0.001
ALT (U/L)	26.6 ± 14.0	29.0 ± 20.2	24.2 ± 12.9	24.9 ± 15.7	28.6 ± 22.1	0.951
*γ*GT (U/L)	26.5 ± 19.6	42.4 ± 56.7	47.5 ± 48.2	68.5 ± 137.1	143.3 ± 117.1	0.001
eGFR (mL/min/1.73 m^2^)	99.8 ± 18.4	75.7 ± 24.0	79.1 ± 29.5	72.1 ± 28.4	61.9 ± 21.5	<0.001
QRS complex, msec	90.3 ± 10.4	93.7 ± 18.1	96.9 ± 19.1	103.3 ± 25.9	110.7 ± 25.6	<0.001
HOP score	−4.50 ± 4.88	1.82 ± 4.42	4.10 ± 5.11	10.77 ± 4.13	18.00 ± 5.02	<0.001
Histidine (*μ*M)	95.2 ± 17.2	88.8 ± 14.3	83.2 ± 16.9	74.1 ± 14.7	71.6 ± 20.5	<0.001
Ornithine (*μ*M)	59.7 ± 19.5	68.7 ± 27.9	77.1 ± 30.5	101.4 ± 36.5	114.8 ± 49.5	<0.001
Phenylalanine (*μ*M)	57.0 ± 8.5	62.3 ± 10.5	66.6 ± 16.7	75.6 ± 18.8	89.0 ± 30.6	<0.001

^∗^Comparison among patients from stages A to D. ACC/AHA: American College of Cardiology/American Heart Association; ACEI: angiotensin-converting enzyme inhibitor; ALT: alanine aminotransferase; ARB: angiotensin receptor blocker; COPD: chronic obstructive pulmonary disease; chronic kidney disease, estimated glomerular filtration rate (eGFR) < 60 mL/min/1.73 m^2^; HF: heart failure; HOP: histidine, ornithine, and phenylalanine; LVEF: left ventricular ejection fraction; *γ*GT: *γ*-glutamyltransferase.

**Table 2:** Comparisons of plasma amino acid concentrations between normal controls and patients at heart failure stage C (Initiation cohort).

		Normal controls	Heart failure stage C	Univariate	Multivariable^‡^	
Amino acid (*μ*M)	VIP score	(*N* = 129)	(*N* = 398)	Odds ratio (95% CI)	Odds ratio (95% CI)	*P* value
Histidine	2.20	95.2 ± 17.2	74.1 ± 14.7^†^	0.92 (0.90–0.94)	0.71 (0.64–0.79)	<0.001
Ornithine	2.09	59.7 ± 19.5	101.4 ± 36.5^†^	1.06 (1.05–1.07)	1.07 (1.04–1.11)	<0.001
Phenylalanine	1.89	57.0 ± 8.5	75.6 ± 18.8^†^	1.13 (1.10–1.16)	1.48 (1.31–1.68)	<0.001
Aspartate	1.28	4.2 ± 2.6	6.7 ± 8.6^†^	1.32 (1.23–1.42)		
Glutamate	1.24	68.3 ± 68.1	137.7 ± 110.5^†^	1.01 (1.009–1.02)		
Glutamine	1.21	679.6 ± 142.5	569.0 ± 173.8^†^	0.995 (0.994–0.997)		
Tyrosine	0.91	65.4 ± 13.1	76.2 ± 24.6^†^	1.03 (1.02–1.04)		
Isoleucine	0.80	72.1 ± 18.6	81.5 ± 23.0^†^	1.02 (1.01–1.03)		
Citrulline	0.78	47.3 ± 27.9	36.3 ± 25.7^†^	0.99 (0.979–0.993)		
Tryptophan	0.63	42.9 ± 12.4	47.6 ± 14.5^†^	1.03 (1.01–1.04)		
Lysine	0.62	216.9 ± 41.0	234.3 ± 58.1^†^	1.06 (1.05–1.07)		
Serine	0.47	126.2 ± 31.1	118.2 ± 31.6^∗^	0.99 (0.98–0.99)		
Arginine	0.41	62.5 ± 21.2	57.4 ± 24.4^∗^	0.99 (0.97–0.99)		
Methionine	0.33	25.3 ± 6.1	23.9 ± 8.5			
Proline	0.33	170.9 ± 66.4	182.5 ± 66.9			
Asparagine	0.25	49.1 ± 27.9	45.9 ± 21.4			
Valine	0.19	243.9 ± 54.5	238.1 ± 58.8			
Leucine	0.11	146.9 ± 41.0	144.5 ± 40.9			
Alanine	0.10	347.7 ± 112.2	340.8 ± 131.1			
Threonine	0.10	116.3 ± 28.6	114.6 ± 33.6			
Glycine	0.09	241.4 ± 72.1	245.3 ± 85.2			

Data are presented as mean ± SD. VIP: variable importance in the projection. ^∗^ *p* < 0.05 and ^†^ *p* < 0.01, compared to normal controls. ^‡^Multivariable analysis was performed for the amino acids with VIP score > 1 by stepwise logistic regression model (forward selection).

**Table 3:** Univariate and multivariable analysis for the HOP score and B-type natriuretic peptide (BNP) for discriminating patients at heart failure stage C from normal controls (initiation cohort).

	Univariate		Multivariable^†^	
	Odds ratio (95% CI)	*p* value	Odds ratio (95% CI)	*p* value
HOP score^∗^	1.53 (1.42~1.66)	<0.001	1.64 (1.45~1.86)	<0.001
Log (BNP) (×10^−2^)	1.05 (1.04~1.06)	<0.001	1.06 (1.04~1.08)	<0.001

^∗^The range of HOP score, value calculated by the combination of histidine, ornithine, and phenylalanine, is from −17.7 to 35.2 (on the basis of logistic regression model). ^†^Multivariable analysis adjusting for age, sex, left ventricular ejection fraction, diabetes mellitus, estimated glomerular filtration rate, and hypertension.

**Table 4:** Cox univariate and multivariable analysis for HF-related rehospitalization and death (initiation cohort).

	Univariate		Multivariable	
Item	Hazard ratio (95% CI)	*p* value	Hazard ratio (95% CI)	*p* value
HOP score	1.072 (1.044~1.100)	<0.001	1.057 (1.024~1.091)	0.001
Log (BNP) × 10^−1^	1.078 (1.045~1.112)	<0.001	1.028 (0.995~1.063)	0.102
Age (years)	1.033 (1.020~1.046)	<0.001	1.029 (1.015~1.043)	<0.001
Sex	0.651 (0.478~0.886)	0.006	0.747 (0.519~1.075)	0.116
LVEF (%)	0.990 (0.980 ~1.000)	0.05	0.989 (0.977~1.001)	0.072
NYHA functional class ≥III	4.196 (1.720~10.233)	0.002	1.455 (0.565~3.748)	0.437
Stage D	1.839 (1.416~2.388)	<0.001	1.417 (1.021~1.088)	0.024
Systolic blood pressure (mmHg)	0.996 (0.988~1.005)	0.402		
Diabetes mellitus	1.685 (1.241~2.288)	0.001	1.360 (0.972~1.902)	0.073
Chronic kidney disease	2.058 (1.503~2.818)	<0.001	1.516 (1.078~2.132)	0.017
Hypertension	1.458 (1.035~2.054)	0.031	1.039 (0.712~1.516)	0.843
Atrial fibrillation	1.274 (0.926~1.751)	0.136		
Body mass index (kg/m^2^)	0.971 (0.935~1.008)	0.119		
Serum sodium (mEq/L)	0.976 (0.936~1.018)	0.189		
Hemoglobin (g/dL)	0.873 (0.814~0.935)	<0.001	0.970 (0.887~1.059)	0.495
Albumin (g/dL)	0.531 (0.409~0.690)	<0.001	0.755 (0.546~1.044)	0.089

BNP: B-type natriuretic peptide; chronic kidney disease, estimated glomerular filtration rate (eGFR) < 60 mL/min/1.73 m^2^; HOP: histidine, ornithine and phenylalanine; LVEF: left ventricular ejection fraction; NYHA: New York Heart Association.

**Table 5:** Demographic and clinical baseline characteristics in different populations defined by the levels of B-type natriuretic peptide (BNP) and HOP score.

	LBLH	HBLH	LBHH	HBHH	
	(*n* = 226)	(*n* = 99)	(*n* = 87)	(*n* = 87)	*p*
Age (years)	59.8 ± 14.5	63.4 ± 13.7	58.5 ± 13.9	63.1 ± 12.2	0.26
Male (%)	151 (66.8)	56 (56.6)	66 (75.9)	56 (64.4)	0.049
LVEF (%)	36.9 ± 15.2	30.2 ± 12.9^†^	36.5 ± 16.4	28.5 ± 11.9^†ϕ^	<0.001
NYHA classification ≥III	216 (95.6)	99 (100)	86 (98.9)	85 (97.7)	0.06
Blood pressure (mm Hg)					
Systolic	123.3 ± 16.9	121.6 ± 19.7	120.2 ± 19.7	122.1 ± 19.3	0.58
Diastolic	74.3 ± 13.1	73.4 ± 12.3	74.3 ± 12.2	74.8 ± 12.5	0.89
Heart rate, beats/min	77.0 ± 11.5	78.1 ± 13.5	77.9 ± 11.9	78.9 ± 11.9	0.63
Comorbidity					
Diabetes mellitus (%)	98 (43.4)	40 (40.4)	32 (36.8)	32 (36.8)	0.62
Hypertension (%)	145 (64.2)	69 (69.7)	44 (50.6)	59 (67.8)	0.034
Atrial fibrillation (%)	80 (35.4)	27 (27.3)	41 (47.1)	21 (24.1)^ϕ^	0.005
COPD (%)	27 (11.9)	8 (8.1)	7 (8.0)	14 (16.1)	0.26
Ischemic (%)	95 (42.0)	51 (51.5)	25 (28.7)	52 (59.8)^∗^^ϕ^	<0.001
Body mass index (kg/m^2^)	26.2 ± 5.5	23.2 ± 3.7^†^	27.9 ± 9.7	24.1 ± 3.9	<0.001
Laboratory data					
Cholesterol (mg/dL)	178.6 ± 45.8	169.5 ± 48.3	160.3 ± 36.7^†^	175.3 ± 42.4	0.009
Triglyceride (mg/dL)	122.9 ± 68.1	115.5 ± 66.1	136.6.9 ± 129.7	124.9 ± 76.4	0.38
LDL-C (mg/dL)	113.6 ± 40.9	106.2 ± 41.7	101.0 ± 31.8^∗^	117.0 ± 31.8^§^	0.016
HDL-C (mg/dL)	41.4 ± 22.6	40.0 ± 15.1	35.2 ± 11.3^∗^	33.3 ± 11.5^†^	0.001
Serum sodium (mEq/L)	139.4 ± 3.2	139.2 ± 2.8	138.5 ± 3.6	137.7 ± 3.8^†‡^	<0.001
Hemoglobin (g/dL)	13.4 ± 2.0	13.0 ± 2.1	13.7 ± 2.3	12.6 ± 2.2^∗^^ϕ^	0.002
Total bilirubin (mg/dL)	1.0 ± 0.6	1.2 ± 0.9	1.3 ± 0.8^∗^	1.5 ± 1.0^†^	<0.001
Albumin (g/dL)	3.7 ± 0.5	3.5 ± 0.4^∗^	3.5 ± 0.5^∗^	3.2 ± 05^†#ϕ^	<0.001
eGFR (mL/min/1.73 m^2^)	77.4 ± 29.2	63.7 ± 25.2^†^	69.5 ± 21.4	58.6 ± 24.9^†§^	<0.001

HB and LB indicate high and low BNP (≥750 and <750 pg/mL, respectively). HH and LH indicate high and low HOP score (≥14 and <14, respectively). COPD: chronic obstructive pulmonary disease; eGFR: estimated glomerular filtration rate; HDL-C: high-density lipoprotein-cholesterol; LDL-C: low density lipoprotein-cholesterol; LVEF: left ventricular ejection fraction; NYHA: New York Heart Association. ^∗^ *p* < 0.05 and ^†^ *p* < 0.01, compared to LBLH; ^‡^ *p* < 0.05 and ^#^ *p* < 0.01, compared to HBLH; ^§^ *p* < 0.05 and *^ϕ^p* < 0.01, compared to LBHH.

## Data Availability

The data used to support the findings of this study are available from the corresponding author upon request.

## Supplementary Materials

Supplementary Materials Supplementary Figure I: study flow diagram. HOP: a metabolic panel composed of histidine, ornithine, and phenylalanine. Supplementary Figure II: the correlation of HOP and B-natriuretic peptide (BNP) to clinical parameters. (A) The relationship between HOP and BNP; (B) a higher blood phenylalanine level correlates with more skeletal muscle loss (∆ skeletal muscle indicates the changes in 14 days after measurement); (C, D) the relationship of HOP and BNP to 6 min walk distance (6MWD). HOP: a metabolic panel composed of histidine, ornithine, and phenylalanine. Supplementary Table I: changes in skeletal muscle mass from baseline to 2 weeks later in patients with different patterns of changes in phenylalanine levels from baseline to 2 weeks later. Supplementary Table II: demographic and laboratory data for the heart failure patients in the validation cohort.
