# Supplementary material for: Amino Acid-Based Metabolic Panel Provides Robust Prognostic Value Additive to B-Natriuretic Peptide and Traditional Risk Factors in Heart Failure
Source: Dis Markers. 2018 Oct 10;2018:3784589. doi: 10.1155/2018/3784589 (PMC6199877; doi:10.1155/2018/3784589)
Supplement: Supplementary Materials — Supplementary Figure I: study flow diagram. HOP: a metabolic panel composed of histidine, ornithine, and phenylalanine. Supplementary Figure II: the correlation of HOP and B-natriuretic peptide (BNP) to clinical parameters. (A) The relationship between HOP and BNP; (B) a higher blood phenylalanine level correlates with more skeletal muscle loss (∆ skeletal muscle indicates the changes in 14 days after measurement); (C, D) the relationship of HOP and BNP to 6 min walk distance (6MWD). HOP: a metabolic panel composed of histidine, ornithine, and phenylalanine. Supplementary Table I: changes in skeletal muscle mass from baseline to 2 weeks later in patients with different patterns of changes in phenylalanine levels from baseline to 2 weeks later. Supplementary Table II: demographic and laboratory data for the heart failure patients in the validation cohort. [file 3784589.f1.docx]

**Supplementary materials**

**Amino acid-based metabolic panel provides robust prognostic value additive to B-natriuretic peptide and traditional risk factors in heart failure**

Chao-Hung Wang, MD, PhD, et al.


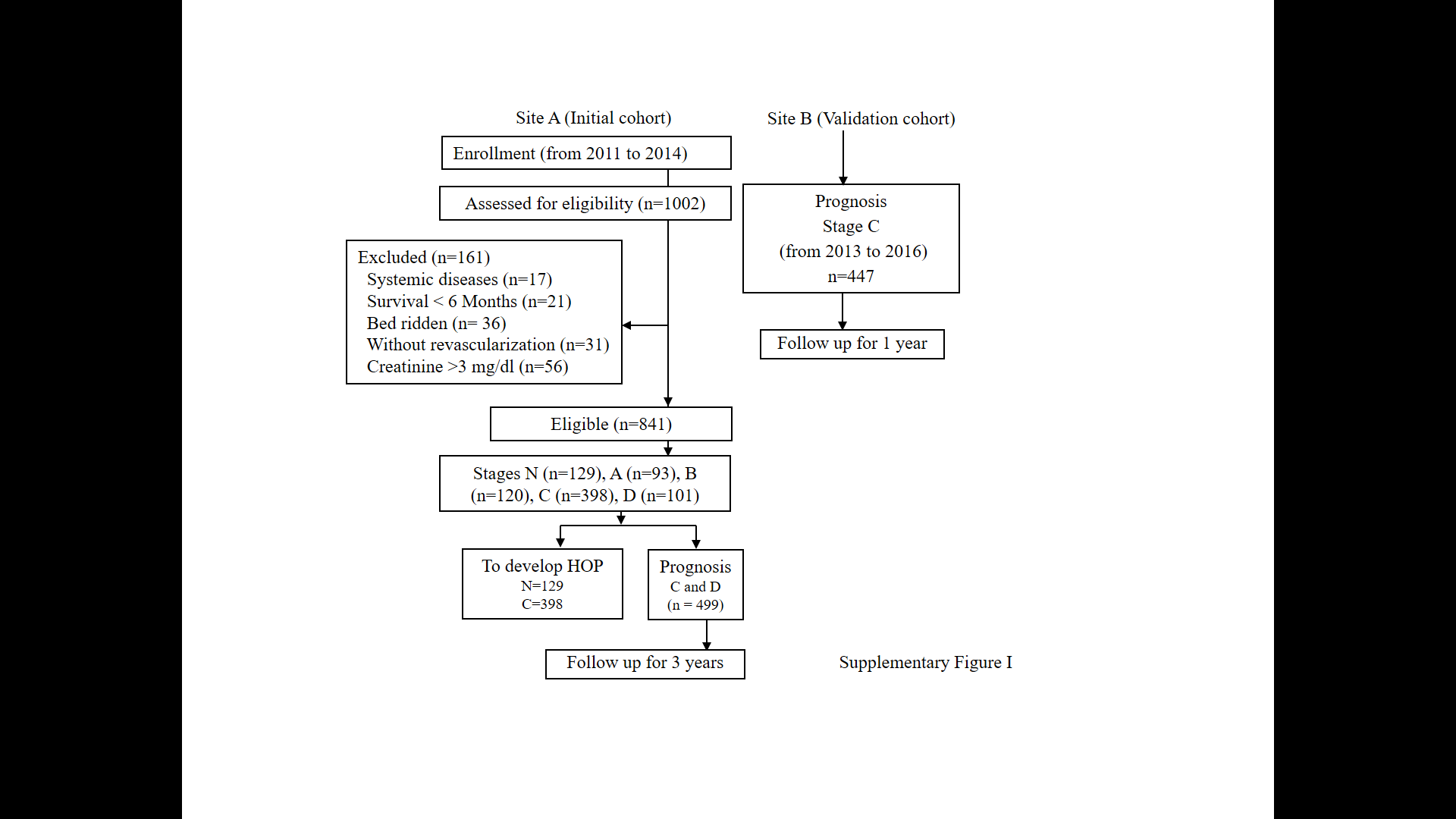


**Supplementary Figure I.** Study flow diagram. HOP, a metabolic panel composed of histidine, ornithine, and phenylalanine.


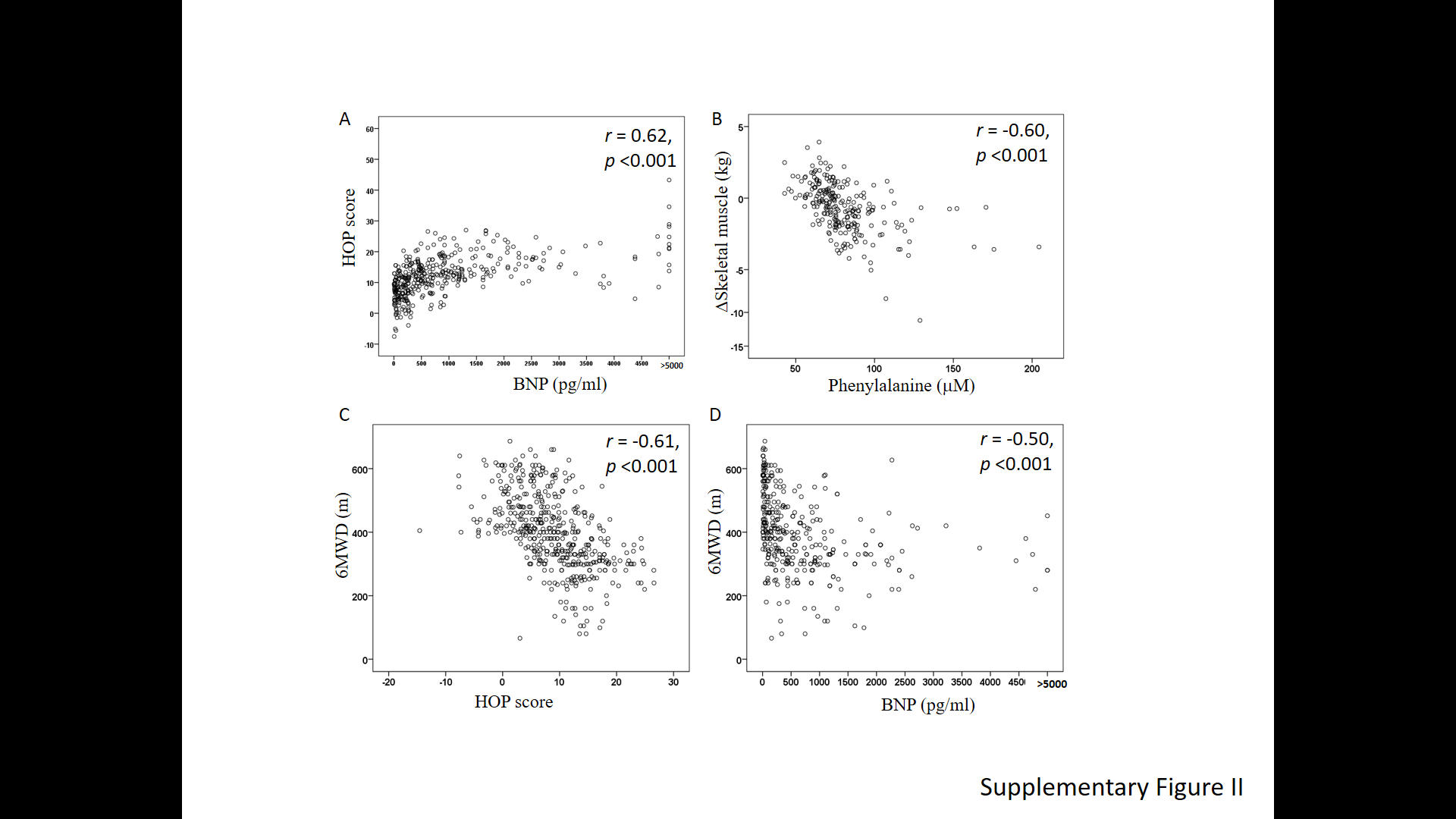


**Supplementary Figure II. The correlation of HOP and B-natriuretic peptide (BNP) to clinical parameters.** A, the relationship between HOP and BNP; B, a higher blood phenylalanine level correlates with more skeletal muscle loss (Δ skeletal muscle indicates the changes in 14 days after measurement); C and D, the relationship of HOP and BNP to 6-min walk distance (6MWD). HOP, a metabolic panel composed of histidine, ornithine, and phenylalanine.

| **Supplementary Table I.** Changes in skeletal muscle mass from baseline to 2 weeks later in patients with different patterns of changes in phenylalanine levels from baseline to 2 weeks later | | | | |  |
| --- | --- | --- | --- | --- | --- |
| Changes in Phenylalanine | high-to-high | high-to-low | low-to-low | low-to-high |  |
|  | n = 39 | n = 9 | n = 48 | n = 5 |  |
| Changes in skeletal muscle mass (kg) from baseline to 2 weeks later | -2.13 ± 2.06 | -0.22 ± 0.72^†^ | 0.39 ± 1.07^†^ | -0.16 ± 0.35* |  |
| “high” and “low” indicate blood phenylalanine levels >69.5 μM and ≤69.5 μM, respectively. **p* <0.05, ^†^*p* <0.01, compared to “high-to-high”. | | | | | |

| **Supplementary Table II.** Demographic and laboratory data for the heart failure patients in the validation cohort | | | |
| --- | --- | --- | --- |
|  |  | Patients |  |
|  |  | n = 447 |  |
| Age (years) |  | 66.9 ± 14.3 |  |
| Male (%) |  | 302 (67.6) |  |
| LVEF (%) |  | 37.9 ± 11.5 |  |
| Co-morbidity |  |  |  |
| Diabetes mellitus (%) |  | 173 (38.7) |  |
| Hypertension (%) |  | 315 (70.5) |  |
| Atrial fibrillation (%) |  | 120 (26.8) |  |
| Ischemia (%) |  | 187 (41.8) |  |
| Laboratory data |  |  |  |
| BNP (ng/ml) |  | 730 ± 1039 |  |
| Log(BNP) |  | 2.37 ± 0.71 |  |
| ALT (U/L) |  | 38.8 ± 83.1 |  |
| γGT (U/L) |  | 38.5 ± 24.2 |  |
| Albumin (g/dl) |  | 3.94 ± 0.54 |  |
| Creatinine (mg/dL) |  | 1.29 ± 1.27 |  |

ALT, alanine aminotransferase; BNP, B-type natriuretic peptide; LVEF, left ventricular ejection fraction; γGT, γ-glutamyltransferase.
